# Comparing Theoretical Models for the Understanding of Health-Risk Behaviour: Towards an Integrative Model of Adolescent Alcohol Consumption

**DOI:** 10.5964/ejop.v16i3.2213

**Published:** 2020-08-31

**Authors:** Andrea Caputo

**Affiliations:** a Department of Dynamic and Clinical Psychology, Sapienza University of Rome, Rome, Italy; Trinity College Dublin, Dublin, Ireland

**Keywords:** alcohol, adolescence, theory of reasoned action, theory of planned behaviour, prototype-willingness model

## Abstract

The aim of the present manuscript is to test and compare the theory of reasoned action (TRA), theory of planned behaviour (TPB) and prototype-willingness model (PWM) in predicting risky alcohol consumption among adolescents and to build an integrative model to get a more comprehensive understanding of such risky behaviour. A total sample of 518 adolescents (55% females; 13-19 aged) recruited from Italian schools (7th to 12th grade) participated in a cross-sectional research study and completed an online questionnaire. Risky alcohol use assessed through the AUDIT-C was the dependent variable; whereas, variables from the TRA, TPB, and PWM (i.e. attitude, subjective norms, perceived behavioural control, intention, prototype favourability and similarity, and willingness to alcohol use) were used as predictors. Data were analysed using structural equation modelling (SEM). The findings show that the integrative model had greater explanatory power and provided a better fit to the data, compared to the TRA, TPB, and PWM, indicating attitudes and subjective norms as the best predictors. In conclusion, perceived social approval from significant others and the volitional component have a central role in understanding adolescents’ alcohol consumption.

Despite the restrictions of policies and laws regulating alcohol consumption in Western countries, alcohol remains one of the most widespread and available drugs used by adolescents (Inchley et al., 2018; Simons-Morton et al., 2009). Along with other risk-taking behaviours, alcohol consumption emerges during early adolescence, often as a means to enhance social relationships with peers when the direct supervision of parents diminishes, then progressing to regular use and increasing the risk of future alcohol dependence (Cleveland, Feinberg, & Jones, 2012; Costello, Sung, Worthman, & Angold, 2007). Indeed, several short- and long-term effects of alcohol can be highlighted, which negatively affect biological, psychological and normal social development, thus contributing to the global burden of disease, injury and economic cost in healthcare (Rehm et al., 2009). Given the vulnerability for alterations in brain structure and function during adolescent age, alcohol may cause neurocognitive, learning and mental health problems (Feldstein Ewing, Sakhardande, & Blakemore, 2014; Marshall, 2014). As well, alcohol consumption may alter social and behavioural functioning, leading to emotional difficulties (Tomlinson, Brown, & Abrantes, 2004), comorbid substance use (Luk et al., 2016), violent conduct and risky sexual behaviours (Komro, Tobler, Maldonado-Molina, & Perry, 2010). Besides this, alcohol can be considered as a risk factor for premature mortality among adolescents in Europe (Inchley et al., 2018) due to its potential harmful and adverse consequences, such as unintentional injuries, road-traffic accidents and suicidal behaviours (Maldonado-Molina, Reingle, Tobler, & Komro, 2010; Swahn, Bossarte, Ashby, & Meyers, 2010).

From this perspective, the need for screening and preventive interventions is advocated to contrast hazardous drinking by adolescents (Liskola et al., 2018), thus requiring a better and deeper understanding of such risky behaviour. Among the existing theoretical models, the theory of reasoned action (TRA; Fishbein & Ajzen, 1975), the theory of planned behaviour (TPB; Ajzen, 1991) and the prototype willingness model (PWM; Gibbons, Gerrard, Blanton, & Russell, 1998) can be conceived as the most popular and fruitful to this purpose.

The TRA assumes that actual behaviour is determined by intentions that reflect the individual’s readiness to perform a given behaviour. Such intentions are in turn a result of two components: attitudes, in terms of overall favourable or unfavourable evaluation of the behaviour, and subjective norms, in terms of perceived approval or disapproval from significant others when performing the behaviour (Fishbein & Ajzen, 1975). The TRA has been used in several studies about adolescent alcohol consumption in both problem (Budd & Spencer, 1984) and non-problem users (Laflin, Moore-Hirschl, Weis, & Hayes, 1994). Despite some studies showing that the TRA succeeded in predicting about 40% of the variance in self-reported drinking among students, with intention being the best predictor (Kuther, 2002; Sheppard, Hartwick, & Warshaw, 1988), other pieces of research found the inadequacy of the TRA components to account the behaviour, depending upon the situational context (Schlegel, Crawford, & Sanborn, 1977) or individual differences (Conner, Rodgers, & Murray, 2007), suggesting the addition of new variables to improve its explanatory power (Lheureux, Auzoult, Charlois, Hardy-Massard, & Minary, 2016). More specifically, the attitude component was associated with alcohol use in adolescents to a higher extent compared to the subjective norm component, which did not add significant prediction to the model, especially when estimating drinking problems (Wood, Nagoshi, & Dennis, 1992). Besides, attitudes and subjective norms resulted to impact the behaviour independent of intentions (Keefe, 1994; Liska, 1984).

The TPB was developed from the TRA (Ajzen, 1991), taking non-volitional behaviours into account by adding perceived behavioural control (PBC) in the model, intended as the individual’s beliefs about the relative ease or difficulty of performing the behaviour and the potential access to necessary resources and opportunities to enact it. PBC is deemed as a third predictor of intentions and as also directly affecting the behaviour to the extent that it accurately reflects the actual control over the behaviour. Previous research studies applying the TPB to alcohol use in adolescent samples found that PBC was the best predictor of problem drinking behaviours in college students (Norman, Bennett, & Lewis, 1998) and added to the predictive power of the model (Rise & Wilhelmsen, 1998). Besides, attitudes only indirectly influenced problem drinking (Wall, Hinson, & McKee, 1998), with positive attitudes being a significant predictor for high school students and negative attitudes being a significant predictor for college ones (Kuther & Higgins-D’Alessandro, 2003). Overall, attitudes and PBC were significant predictors of intentions to use alcohol, which in turn impacted actual drinking behaviours (Mcmillan & Conner, 2003). However, the studies comparing the predictive ability of the TRA to the predictive ability of the TPB showed conflicting results. Some pieces of research concluded that the TPB was more predictive of problem drinking, with PBC directly affecting alcohol consumption only in problem drinkers (Schlegel, D’Avemas, Zanna, DeCourville, & Manske, 1992); whereas, TRA was superior to the TPB in predicting alcohol use (Laflin et al., 1994). Some studies found that PBC and subjective norms were not useful in predicting college students’ intentions to consume alcohol (Trafimow, 1996). Instead, other studies concluded that TPB was better in predicting intentions to alcohol consumption and, more specifically, PBC was the most influential factor (Marcoux & Shope, 1997).

As both the TRA and TPB were criticised for their assumption that behaviours are mostly rational and planned, without considering the role of unconscious or heuristic processes (Sheeran, Gollwitzer, & Bargh, 2013), the PWM was developed to predict more spontaneous risky behaviours among adolescents (Gibbons, Gerrard, Blanton, & Russell, 1998). Specifically, the PWM assumes two different pathways (the reasoned and the social reactive path) to explain the behaviour. The reasoned pathway includes attitudes and subjective norms as predictors of intentions, which in turn impact the actual behaviour as in the TRA. Instead, the social reactive pathway includes prototypes, as images of a typical person engaging in the behaviour in terms of perceived favourability and similarity of the prototype to the individual, and willingness, as the individual’s tendency to perform a behaviour when s/he has an opportunity in situations that are conducive to that behaviour. The prototypes affect the willingness, which in turn affects intentions and directly predicts the behaviour. The PWM has been used to predict health-risk behaviours in adolescents, especially alcohol consumption (Andrews, Hampson, Barckley, Gerrard, & Gibbons 2008; Gerrard et al., 2002; Todd, Kothe, Mullan, & Monds, 2016), showing that prototype favourability and similarity were associated with behaviour, intentions and willingness with small-to-medium effect sizes (van Lettow, de Vries, Burdorf, & van Empelen, 2016). The prototype perceptions were demonstrated to account for both binge drinking intentions and behaviour, although only prototype similarity emerged as a significant predictor (Norman, Armitage, & Quigley, 2007). Overall, research suggested that the PWM variables, especially prototype similarity, can enhance the predictive validity of the TPB in explaining behavioural intentions to risky behaviours (Rivis, Sheeran, & Armitage, 2006).

Based on the discussed literature, the aim of the present study is to test and compare the TRA, TPB, and PWM in predicting risky alcohol consumption among adolescents. As well, in line with more recent studies suggesting the usefulness of blending the elements of such different theories (Demir, Özkan, & Demir, 2019; Frater, Kuijer, & Kingham, 2017; Rivis et al., 2006), we aim at building an integrative model (Figure 1) including the most significant predictors of alcohol consumption, so to get a more comprehensive and suitable framework for the understanding of such risky behaviour.

**Figure 1:**
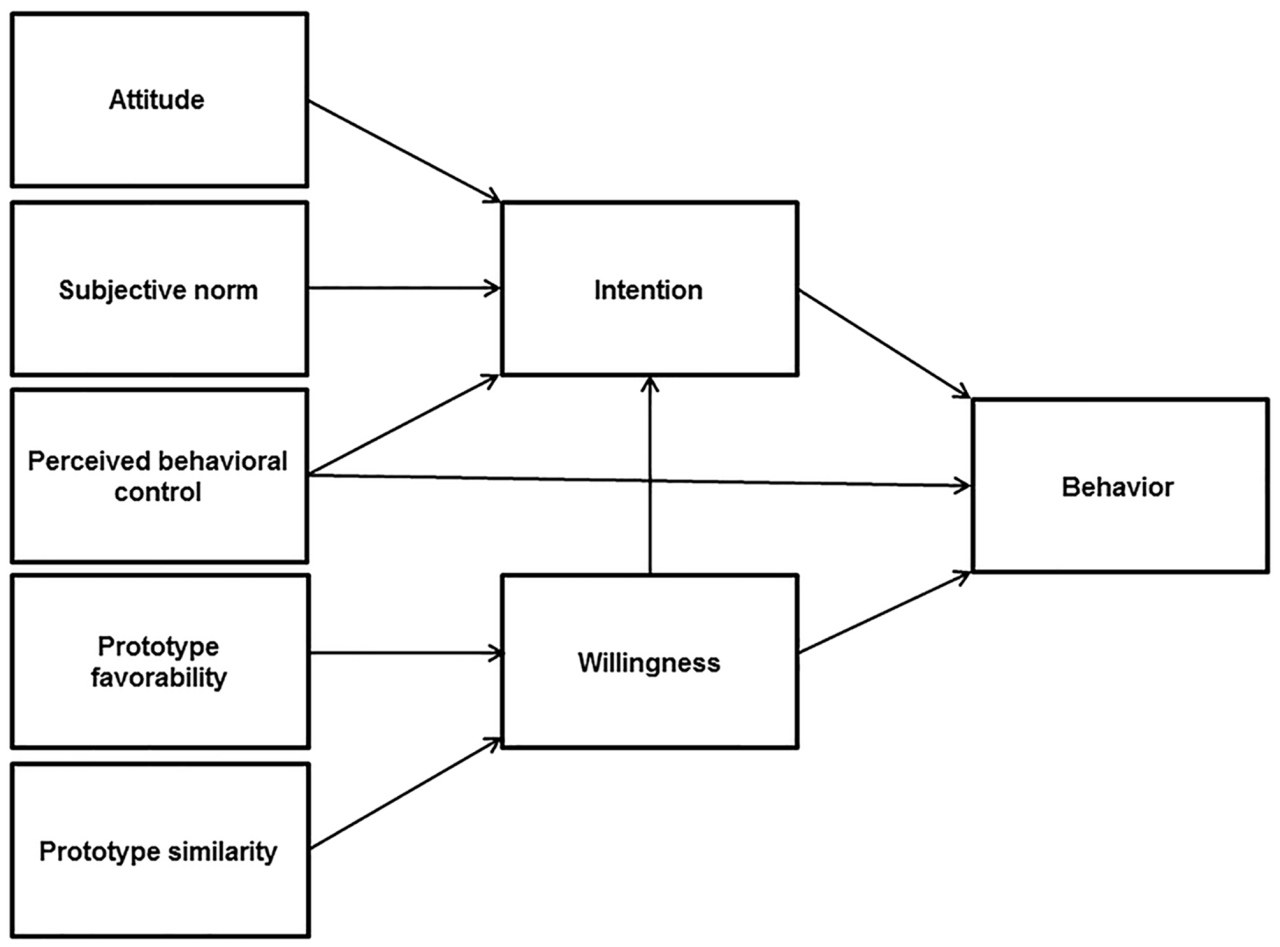
The integrative model (adapted from Demir, Özkan, & Demir, 2019).

## Materials and Method

### Participants

The target population for this cross-sectional research study included students enrolled in lower and secondary schools of Rome, participating in a research project about adolescent alcohol use on a voluntary base. The total sample consisted of 518 students from 7th to 12th grade (55% females, 45% males) 13-19 aged (*M* = 15.39, *SD* = 1.43), who were Italian in 81.5% of cases. For minor participants, informed written consent was given by both parents. All students provided their informed consent to participate in the study before completing an online questionnaire, which included basic demographics, risky alcohol use measures, and variables from the TRA, TPB, and PWM.

### Measures

#### Risky Alcohol Use

The Italian version of AUDIT-C (Alcohol Use Disorders Identification Test – Consumption; Struzzo, De Faccio, Moscatelli, & Scafato, 2006) was used as a measure of hazardous and harmful alcohol consumption. AUDIT-C is based on the first three items of AUDIT developed by the World Health Organisation and refers to the frequency of alcohol consumption, the number of standard drinks consumed on a typical day when drinking, and the frequency of consuming six or more standard drinks on one occasion. It estimates alcohol consumption in a standard, meaningful and non-judgmental manner and allows the detection of at-risk drinking habits in the adolescent population significantly better than the entire AUDIT (DeMartini & Carey, 2012). AUDIT-C uses a five-point Likert scale, with 0 being no problem drinking and 4 being problematic, and has an overall score ranging from 0 to 12. The Cronbach’s alpha for the present study was .75.

#### Attitudes

To measure attitudes toward alcohol consumption, five semantic differential items were adapted from previous studies (Gibbons et al., 1998; Quigley, 2010; Zimmermann & Sieverding, 2010). The participants completed the sentence ‘‘For me, consuming alcohol in the next month would be. . .” with responses ranging from 1 to 7 (*negative* to *positive*, *boring* to *amusing*, *unpleasant* to *pleasant*, *harmful* to *beneficial*, and *unhealthy* to *healthy*) and higher averaged scores indicating more positive attitude. The Cronbach’s alpha for the present study was .90.

#### Subjective Norms

To measure subjective norms two items derived from previous studies were used (Gibbons et al., 1998; Kalebić Maglica, 2011; Quigley, 2010; Zimmermann & Sieverding, 2010) regarding social approval about alcohol consumption by parents and friends (“How do you think your parents [friends] would respond if they thought you consume alcohol?”), followed by a 7-point bipolar scale (from 1 = *definitely disapprove* to 7 = *definitely approve*). Scores were averaged to provide a measure of subjective norms, with higher scores indicating greater perceived approval from significant others.

#### Perceived Behavioural Control

Perceived behavioural control was assessed with two items derived from previous studies (Gibbons et al., 1998; Quigley, 2010) assessing the extent to which the respondent feels like s/he has the ability to consume alcohol or not based on personal will (“If I wanted to consume alcohol, I could easily do it” and “If I did not want to consume alcohol, I would be able to control myself”). The items were assessed on a 7-point Likert scale ranging from 1 (*strongly disagree*) to 7 (*strongly agree*). The mean from the two items provided a measure of perceived behavioural control, with higher scores indicating greater self-efficacy.

#### Intention

The participants’ intention to consume alcohol was measured with two items derived from previous studies (Gibbons et al., 1998; Quigley, 2010; Zimmermann & Sieverding, 2010) and assessed on a 7-point likert scale as follows: “Do you intend to drink to get drunk over the next month?” (*definitely do not*-*definitely do*) and “How likely are you to drink to get drunk in the next month?” (*very unlikely*-*very likely*). The mean from the two items provided a measure of intention, with higher scores indicating a positive intention to drinking to get drunk in the next month.

#### Prototype Perceptions

Prototype favourability was measured by asking “Please think about the typical person your age who drinks alcohol. How much do you think the following words describe your image of that person?” (Gerrard et al., 2002; Litt & Stock, 2011). Following the stem were 3 different adjectives (smart, popular, attractive), each rated on a scale from 1 (*not at all*) to 7 (*extremely*). Whereas, prototype similarity was measured using the same 3-item scale by asking: ‘‘How similar are you in general to this type of person based on such characteristics?”, with each adjective rated on a scale from 1 (*not at all similar*) to 7 (*very similar*). The means from the items respectively provided a measure of positive opinion about a peer consuming alcohol and assumed similarity with such a prototype, with higher scores indicating greater favourability/similarity perceptions. The Cronbach’s alpha for the present study was .87 and .89, respectively.

#### Willingness

Willingness to consume alcohol was assessed by a scale used in previous studies (Gibbons et al., 1998; Kalebić Maglica, 2011; Litt & Stock, 2011; Quigley, 2010; Zimmermann & Sieverding, 2010) asking participants to imagine themselves with some friends where one of those friends offered them alcohol. This was followed by three questions respectively asking participants how likely it would be that they would: 1) accept alcohol, 2) say “no thanks” and refuse alcohol, and 3) leave the situation. Such possible reactions were measured on a 7-point scale ranging from 1 (*not at all likely*) to 7 (*extremely likely*). Responses to the negatively worded items were reverse-scored and the items were then averaged, with higher scores indicating greater willingness. The Cronbach’s alpha for the present study was .67.

### Statistical Analyses

The descriptive statistics and Pearson’s bivariate correlations among the study variables were analysed. Then, path analyses for risky alcohol consumption from the TRA, TPB, PWM and the integrative model were conducted through Structural Equation Modelling (maximum likelihood method) using STATA (Release 14.0). To evaluate the model fits, the following indices were used: the χ^2^ ratio (χ^2^ /degrees of freedom, *df*), RMSEA, SRMR, CFI, and TLI. Smaller χ^2^ value corresponds to better-fitting models (Schumacker & Lomax, 2010). As Hu and Bentler (1999) stated, RMSEA values up to .06 and SRMR values up to .08 are indicative of good fit; whereas CFI and TLI values higher than .95 generally indicate good model fit. Besides this, the standardised path coefficients and *R*^2^ values were taken into account to assess the predictive power and the robustness of the models. When testing the integrative model, the modification indices were calculated to include (or omit) only the suggested paths that were theoretically sensible.

## Results

Descriptive statistics and correlations among the study variables are presented in Table 1, revealing the presence of statistically significant associations among all the examined measures with small-to-medium effect sizes, except for the relationship between prototype similarity and willingness (*r* = .08, *p* = .079).

**Table 1:** Descriptive Statistics and Bivariate Correlations Among the Study Variables (N = 518)

Measure	*M*	*SD*	1	2	3	4	5	6	7	8
1. At-risk alcohol use	2.07	1.94								
2. Attitudes	3.40	1.63	.34**							
3. Subjective norms	3.23	1.58	.47**	.41**						
4. Perceived behavioural control	4.85	1.63	.32**	.31**	.47**					
5. Intention	3.18	1.97	.46**	.37**	.41**	.35**				
6. Prototype favourability	2.25	1.52	.26**	.21**	.24**	.14*	.29**			
7. Prototype similarity	2.96	1.70	.21**	.19**	.22**	.19**	.18**	.43**		
8. Willingness	4.23	1.62	.38**	.34**	.37**	.26**	.43**	.11*	.08	

*p < .01. **p < .001.

The path analyses for each examined model (Table 2) are presented as follows.

**Table 2:** Direct and Indirect Effects in Path Models of At-Risk Alcohol Use (Standardised Coefficients, N = 518)

Path model	Intention			Willingness			Behaviour		
	Direct	Indirect	Total	Direct	Indirect	Total	Direct	Indirect	Total
TRA									
Attitudes	.30**		.30**					.14**	.14**
Subjective norms	.38**		.38**					.17**	.17**
Intention							.45**		.45**
TPB									
Attitudes	.27**		.27**					.11**	.11**
Subjective norms	.30**		.30**					.12**	.12**
PBC	.21**		.21**				.21**	.08**	.29**
Intention							.39**		.39**
PWM									
Attitudes	.22**	.08**	.30**	.22**		.22**		.17**	.17**
Subjective norms	.28**	.10**	.38**	.29**		.29**		.22**	.22**
Prototype favourability		.01	.01	.02		.02		.01	.01
Prototype similarity		-.01	-.01	-.04		-.04		-.01	-.01
Willingness	.35**		.35**				.26**	.12**	.38**
Intention							.36**		.36**
Integrative model									
Attitudes	.20**	.07**	.27**	.22**		.22**		.11**	.11**
Subjective norms	.21**	.10**	.31**	.29**		.29**	.38**	.13**	.51**
PBC	.19**		.19**					.05*	.05*
Willingness	.33**		.33**				.17**	.09**	.26**
Intention							.27**		.27**

*Note.* TRA = Theory of reasoned action. TPB = Theory of planned behaviour. PBC = Perceived behavioural control. PWM = Prototype-willingness model.

*p < .01. **p < .001.

**Figure 2:**
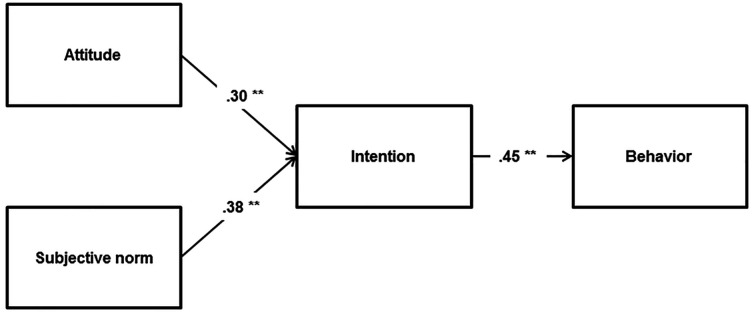
The TRA for risky alcohol consumption. **p < .001.

The TRA did not provide a good fit to the data (χ*^2^*/*df* = 73.067/2, RMSEA = .262, SRMR = .087, CFI = .778, TLI = .446). The results indicated that intention was significantly predicted by attitudes (β = 0.25, *p* < .001) and subjective norms (β = 0.31, *p* < .001); as well, behaviour was also predicted by intention (β = 0.46, *p* < .001). The model explained 22% and 21% variance in intentions and behaviours, respectively (Figure 2).

**Figure 3:**
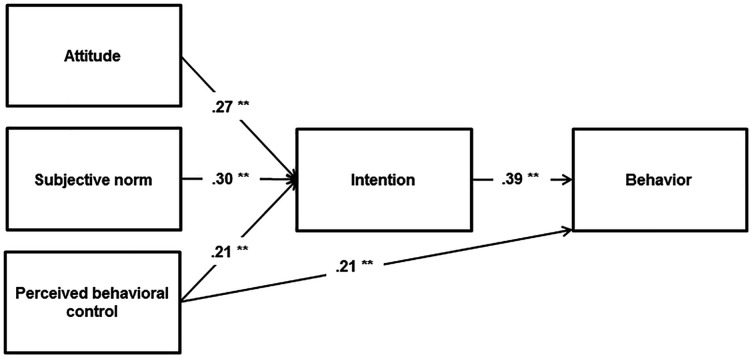
The TPB for risky alcohol consumption. **p < .001.

The TPB did not provide a good fit to the data (χ^2^/*df* = 56.419/2, RMSEA = .229, SRMR = .059, CFI = .838, TLI = .432). The results indicated that intention was significantly predicted by attitudes (β = 0.22, *p* < .001), subjective norms (β = 0.24, *p* < .001) and PBC (β = 0.17, *p* < .001); as well, behaviour was also predicted by intention (β = 0.40, *p* < .001) and PBC (β = 0.17, *p* < .001). The model explained 24% variance in both intentions and behaviours, respectively (Figure 3).

**Figure 4:**
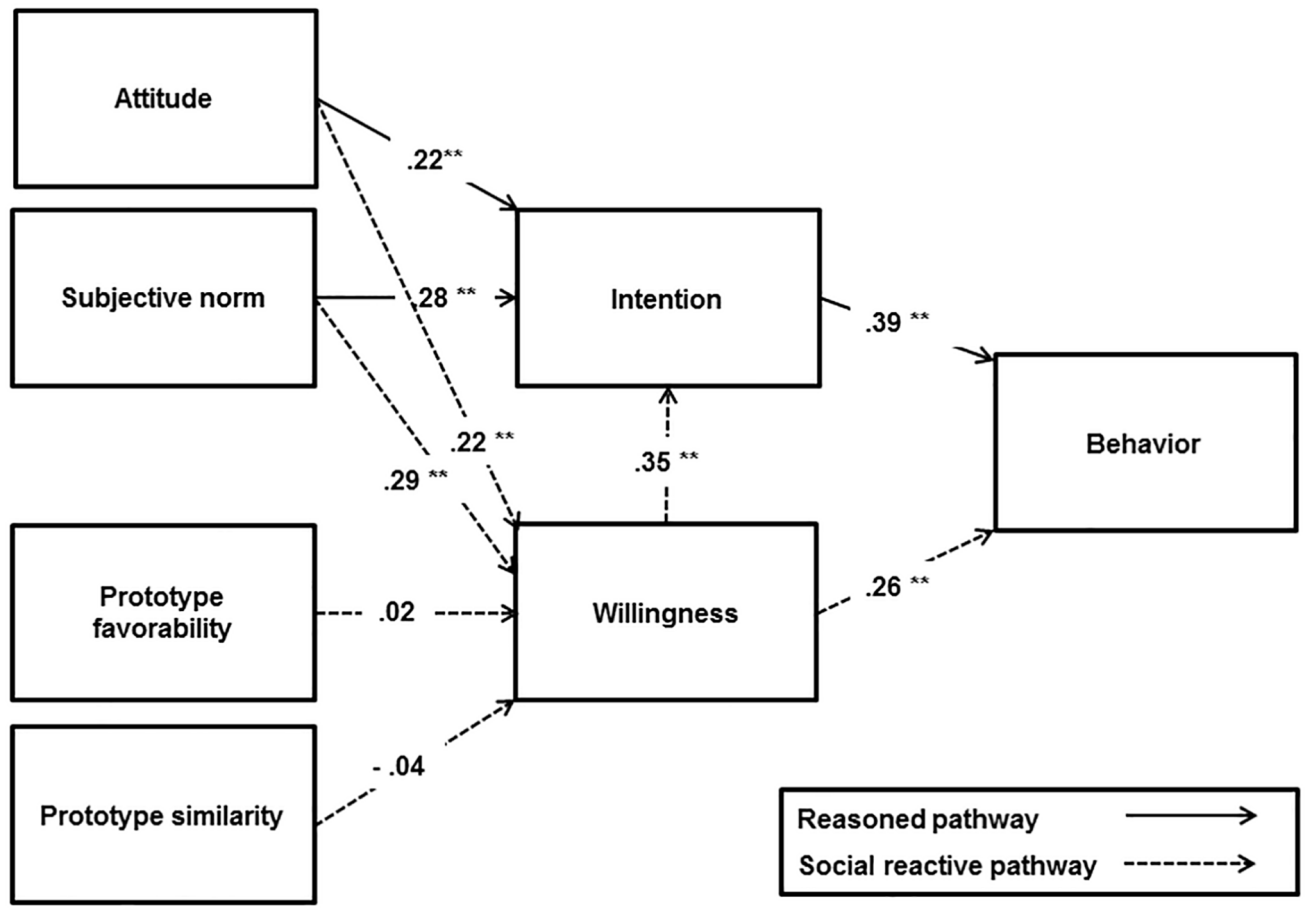
The PWM for risky alcohol consumption. **p < .001.

The PWM did not provide a good fit to the data (χ^2^/*df* = 86.686/6, RMSEA = .161, SRMR = .069, CFI = .839, TLI = .596). The results indicated that intention was significantly predicted by attitudes (β = 0.19, *p* < .001), subjective norms (β = 0.23, *p* < .001) and willingness (β = 0.28, *p* < .001); as well, behaviour was also predicted by intention (β = 0.37, *p* < .001) and willingness (β = 0.22, *p* < .001). The explained variance in intentions, willingness, and behaviours were 29%, 18%, and 25%, respectively (Figure 4).

**Figure 5:**
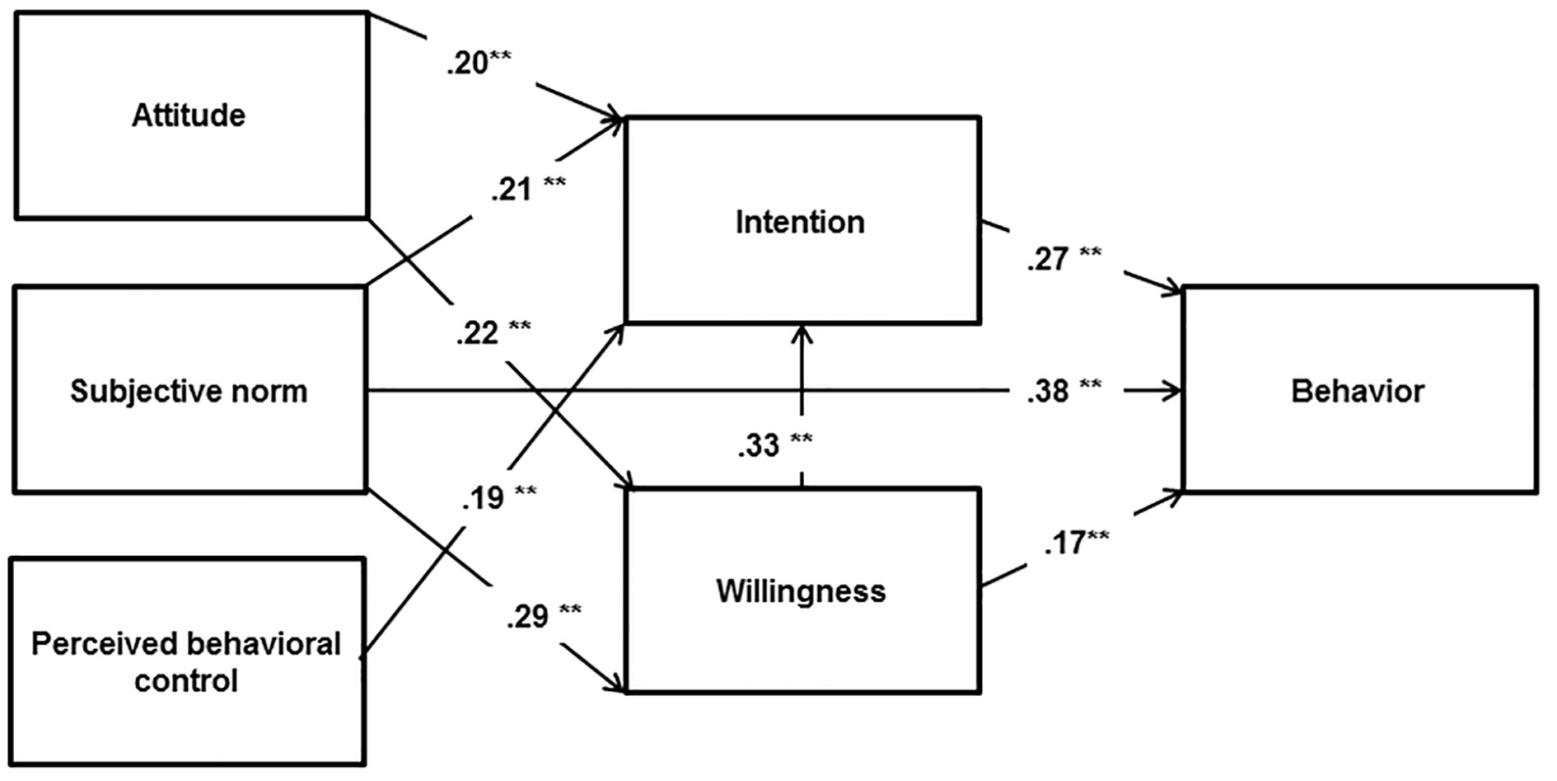
The integrative model for risky alcohol consumption. **p < .001.

The integrative model did not provide a good fit to the data (χ^2^/*df* = 172.986/9, RMSEA = .188, SRMR = .108, CFI = .681, TLI = .362). Thus, the modification indices were evaluated and some suggested theoretically reasonable paths were included in the model, as follows: omitting the paths from prototype favourability and similarity to willingness, including the paths from attitudes and subjective norms to willingness, omitting the path from PBC to behaviour and including the path from subjective norms to behaviour. After the modifications, the model provided a good fit to the data (χ^2^/*df* = 7.635/3, RMSEA = .055, SRMR = .019, CFI = .991, TLI = .962), and explained a satisfactory percentage of variance in intentions (30%), willingness (18%), and behaviours (32%) (Figure 5).

## Discussion

The present study aimed at testing and comparing the TRA, TPB, and PWM as the most popular conceptual models able to predict risky alcohol consumption among adolescents, as well as at building an integrative model blending different elements of such theories to get a more comprehensive and suitable framework for the understanding of such risky behaviour.

The TRA explained 22% of the variance in intentions to alcohol consumption and 21% variance in risky drinking behaviour. Among the TRA constructs, subjective norms emerged as the most important predictor of the intention in line with meta-analytic findings by Cooke, Dahdah, Norman, and French (2016), thus confirming the relevant role of expectancies of the social environment and significant others in determining health risk behaviours. As well, behavioural intention was found to be the main motivator of behaviour consistently with previous research (Cooke et al., 2016; Kuther, 2002; Sheppard et al., 1988), suggesting the volitional nature of alcohol consumption among adolescents.

The TPB, including the PBC component, enhanced the predictive power of the model only to a small extent, adding 2% and 3% for the explanation of alcohol consumption related intentions and behaviours, confirming what found by other studies on drinking behaviour (Marcoux & Shope, 1997; Schlegel et al., 1992). As well, only 27.6% of the effect of PBC on the behaviour was mediated by intentions, deemphasising the central role of the individual’s perceived control over the ability to engage in alcohol consumption, which may lead to unhealthy behaviours. Some possible explanations can be provided for the scarce increase of the predictive power of the TPB model including the PBC component. For instance, actual alcohol access and use could be determined (to some extent) by factors beyond adolescents‘ control and thus PBC may not be reasonably realistic (Ajzen & Madden, 1986). Besides, PBC could be most relevant in goal-oriented behaviours that require plans of action or implementation intentions (Gibbons, Houlihan, & Gerrard, 2009), as in the case of health-promoting behaviours. This is confirmed by a previous meta-analysis of 206 TPB prospective studies (McEachan, Conner, Taylor, & Lawton, 2011), showing that PBC is overall more effective at predicting health-promoting behaviours (e.g., physical activity, safer sex, and dietary behaviours), rather than health-risk ones (e.g., drinking alcohol, smoking, using drugs, speeding).

The results about the PWM testing, which added 7% and 4% to the prediction of intentions and behaviours compared to the TRA (considered as a baseline), showed that the social reactive path was substantially less relevant in explaining alcohol consumption, thus suggesting that some use is planned and intentional even for young adolescents (Andrews et al., 2008; Webb, Ashton, Kelly, & Kamali, 1996). Specifically, prototype perceptions in terms of favourability and similarity did not have any effect on willingness, thus revealing that opportunities to engage in risky behaviours are independent of the characteristics that adolescents associate with the prototypical drinker individual. This seems consistent with previous experimental research findings showing that prototype manipulation did not affect alcohol consumption (Todd & Mullan, 2011). As well, this could depend on the specific measure used in the present study to assess prototype perceptions, because recent research has shown that more “extreme” drinker prototypes (heavy drinker or abstainer prototypes) are most predictive of alcohol consumption among adolescents (Teunissen, Spijkerman, Kuntsche, Engels, & Scholte, 2017). However, willingness was found to be the best predictor of intentions, despite its direct effect on the behaviour was lower than that of intentions.

As none of the examined theoretical frameworks provided a good fit to the data, an integrative model was build and tested from what suggested by previous research studies (Demir et al., 2019; Frater et al., 2017; Rivis et al., 2006). Such a model showed good fit indices and succeeded to explain a satisfactory percentage of variance in intentions (30%) and behaviours (32%), respectively adding further 8% and 11% to the prediction of such outcomes compared to the TRA. The model included attitudes, subjective norms, PBC and willingness as predictors of intentions to alcohol consumption, with willingness having the strongest effect. Willingness was predicted by attitudes and subjective norms; whereas, intentions, subjective norms, and willingness impacted the behaviour, with subjective norms having the strongest total effect on alcohol consumption as found in a research study about safe sexual practice (Trafimow, 1994).

Overall, consistently with previous findings on adolescents’ drinking behaviours (Blanton, Gibbons, Gerrard, Conger, & Smith, 1997; Litt & Stock, 2011), the perceived social approval from significant others seems to be a meaningful factor, given its influence on alcohol consumption (also mediated by intentions and willingness), thus suggesting the importance of social environment and influences for alcohol prevention (Neighbors, Lee, Lewis, Fossos, & Larimer, 2007). As well, the role of positive attitudes towards alcohol in determining greater alcohol consumption through enhancing intentions, and willingness should be considered to reinforce the advantages of the target behaviour and address barriers. An interesting result about the examined integrative model concerns the lack of a direct effect of PBC on risky drinking, despite PBC indirectly influencing it through intentions, which may be probably due to the greater alcohol availability among adolescents and opportunities for consumption in current times thus making alcohol use more volitional (Fishbein & Ajzen, 2010; Glassman, Braun, Dodd, Miller, & Miller, 2010; Jamison & Myers, 2008). This seems to be also confirmed by the relevant role of intentions to perform risky drinking according to a reasoned pathway, although willingness in terms of spontaneous and reactive decision‐making may contribute to enhance intentions to a significant extent. Indeed, previous studies found that intentions represent a better predictor (compared to willingness) for adolescents who are experienced with the behaviour (Gerrard, Gibbons, Houlihan, Stock, & Pomery, 2008; Pomery, Gibbons, Reis-Bergan, & Gerrard, 2009).

Some limitations should be acknowledged regarding the present study, such as the convenience nature of the sample that does not consent any generalisation, the lack of a longitudinal design that could improve the model testing performance and the presence of potential confounders such as age and gender that could have biased the results. In this regard, future research studies could rely on longitudinal designs, incorporating previous alcohol consumption from adolescents as antecedent potentially affecting intentions and behaviours. Besides, subgroup analyses by gender and age levels could further disentangle the interrelations among the study variables and provide more accurate conceptual frameworks for the understanding of risky drinking and more specific indications for prevention and treatment. Another limitation refers to the lack of social desirability measures, which have been demonstrated to enhance self-reported wellbeing (Caputo, 2017). Indeed, socially desirable responding (e.g., impression management, self-deception) should be carefully considered, especially when assessing unhealthy behaviours like drug or alcohol use (Caputo, 2019a, 2019b). In this regard, alcohol consumption may be under-reported since it is not socially acceptable, because in Italy it is illegal to sell alcoholic beverages to minors and any alcohol use among them should be avoided according to the National Institute of Health (Istituto Superiore di Sanita - ISS).

Besides, since the sample was almost entirely composed of Italian respondents (81.5%), the role of some contextual factors should be considered to put some of the present findings into perspective. Indeed, cultural models (in terms of shared representations about a phenomenon) may orient attitudes, interactions, and practices that are socially accepted within a specific context (Caputo, 2013a). For instance, the relevance of perceived social approval found in the current study seems to be consistent with previous cross-cultural research (Petrilli, Beccaria, Prina, & Rolando, 2014; Rolando & Beccaria, 2018), highlighting the role of social values that emphasise family ties and the sharing of informal norms in the Italian alcohol socialization process. As well, the strong predictive role of positive attitudes appears in line with the reduced stigmatisation of drinking among Italian young people (Petrilli et al., 2014; Rolando, Beccaria, Tigerstedt, & Törrönen, 2012), that is typical of Mediterranean countries where alcohol is used predominantly for nutrition (Savic, Room, Mugavin, Pennay, & Livingston, 2016). Then, the hypothesised volitional nature of actual alcohol use in our sample seems consistent with the negative attitude towards alcohol intoxication found among adolescents in Italy, compared to the USA and northern Europe (Rolando & Beccaria, 2018; Savic et al., 2016). Indeed, Italian adolescents are generally more conscious about possible risks of drinking (Rolando & Beccaria, 2018; Savic et al., 2016) and, accordingly, drunkenness may be viewed as the result of a voluntary decision.

In conclusion, the current study suggests that the TRA, TPB, and PWM seem to be quite similar in terms of predictive power and do not represent completely accurate and robust conceptual models for the understanding of risky drinking among adolescents. Specifically, the PWM appears as partially inadequate given the lack of associations between prototype perceptions and willingness. However, the development of an integrative model blending different but complementary elements from the TRA, TPB, and PWM may constitute a more comprehensive framework, whose preliminary results indicate the central role of subjective norms and the greater salience of volitional components in understanding adolescents’ alcohol consumption.

Overall, the presented integrative model sheds light on the prominent role of perceived social approval from significant others, positive attitudes towards alcohol, and intentions to perform risky drinking among adolescents. Accordingly, some practical implications can be thus derived from the present study, which may have a meaningful role for prevention, and risk mitigation actions and policies. About perceived social approval, this study suggests parent participation in alcohol prevention. Indeed, parents are important facilitators for the transmission of alcohol-related attitudes and rules (Adolfsen et al., 2017), and the perceived legitimacy of parental authority regarding alcohol use is a relevant factor during early adolescence (Jackson, 2002). As well, because of the strong association between peer alcohol use and individual drinking habits in student populations (Kuntsche & Jordan, 2006), intervention programs should be carried out at the class and school level, thus overall contributing to better organisational health (Caputo & Rastelli, 2014). Specifically, such interventions could promote peer education and group discussion about alcohol-related issues, so that peers may act as positive role models who can reinforce behavioural messages (MacArthur, Harrison, Caldwell, Hickman, & Campbell, 2016). Concerning attitude towards alcohol, drinking-related meanings and motives should be further explored and worked on to better understand their positive connotation. As suggested by previous studies on adolescent alcohol use and other problem behaviours (Cooper, 1994; Langher et al., 2019), drinking could be triggered by several underlying motives, such as coping with negative experiences (that is considered as the most dysfunctional one). Deepening such motives may thus help adolescents understand and reflect on real-life difficulties and challenges that may lead to alcohol use, “as the specific meaning of health risks and benefits is related to the individuals’ processes of adaptation in different life (family, work, social) contexts” (Caputo, 2013b, p. 153). Then, the volitional nature of risky alcohol use seems to suggest that adolescents are aware of the risks of drinking to some extent. From this perspective, it is important to consider them as competent individuals and avoid taking a paternalistic tone in education and prevention initiatives (Rolando & Beccaria, 2018). Whereas, the beliefs about what they expect to experience as a result of drinking should be taken into account, such as enhancing positive mood, conforming to peer pressure or improving social interactions, providing them alternative and protective behavioural strategies (Reid & Carey, 2015).
